# The BrAID study protocol: integration of machine learning and transcriptomics for brugada syndrome recognition

**DOI:** 10.1186/s12872-021-02280-3

**Published:** 2021-10-13

**Authors:** M. A. Morales, M. Piacenti, M. Nesti, G. Solarino, P. Pieragnoli, G. Zucchelli, S. Del Ry, M. Cabiati, F. Vozzi

**Affiliations:** 1 CNR Institute of Clinical Physiology, Via Giuseppe Moruzzi 1, 56124 Pisa, Italy; 2 Fondazione Toscana Gabriele Monasterio, Via G. Moruzzi 1, Pisa, Italy; 3 U.O.C. Cardiologia Ospedale San Donato, Via Pietro Nenni 20, Arezzo, Italy; 4 Azienda Usl Toscana Nord Ovest U.O.C. Cardiologia Ospedale Versilia, SS1 Via Aurelia 335, Lido di Camaiore, Italy; 5 Azienda Ospedaliera Universitaria Careggi SOD Aritmologia, Largo Brambilla, 3, Firenze, Italy; 6 Azienda Ospedaliero Universitaria Pisana Cardiologia 2 U.O.C. Cisanello, Via Paradisa, 2, Pisa, Italy

**Keywords:** Brugada syndrome, Machine learning, Transcriptomic, RNA

## Abstract

**Background:**

Type 1 Brugada syndrome (BrS) is a hereditary arrhythmogenic disease showing peculiar electrocardiographic (ECG) patterns, characterized by ST-segment elevation in the right precordial leads, and risk of Sudden Cardiac Death (SCD). Furthermore, although various ECG patterns are described in the literature, different individual ECG may show high-grade variability, making the diagnosis problematic. The study aims to develop an innovative system for an accurate diagnosis of Type 1 BrS based on ECG pattern recognition by Machine Learning (ML) models and blood markers analysis trough transcriptomic techniques.

**Methods:**

The study is structured in 3 parts: (a) a retrospective study, with the first cohort of 300 anonymized ECG obtained in already diagnosed Type 1 BrS (75 spontaneous, 150 suspected) and 75 from control patients, which will be processed by ML analysis for pattern recognition; (b) a prospective study, with a cohort of 11 patients with spontaneous Type 1 BrS, 11 with drug-induced Type 1 BrS, 11 suspected BrS but negative to Na + channel blockers administration, and 11 controls, enrolled for ECG ML analysis and blood collection for transcriptomics and microvesicles analysis; (c) a validation study, with the third cohort of 100 patients (35 spontaneous and 35 drug-induced BrS, 30 controls) for ML algorithm and biomarkers testing.

**Discussion:**

The BrAID system will help clinicians improve the diagnosis of Type 1 BrS by using multiple information, reducing the time between ECG recording and final diagnosis, integrating clinical, biochemical and ECG information thus favoring a more effective use of available resources.

*Trial registration* Clinical Trial.gov, NCT04641585. Registered 17 November 2020, https://clinicaltrials.gov/ct2/show/NCT04641585

## Background

Brugada Syndrome (BrS) is a hereditary arrhythmogenic disease with an electrocardiographic (ECG) pattern characterized by ST-segment elevation in the right precordial leads and burdened by the risk of sudden cardiac death [1]. The prevalence of the syndrome is estimated to be 15 per 10,000 in South East Asia, including Japan, and 2 per 10,000 in the Western countries. BrS has been considered responsible for 4–12% of all sudden deaths and up to 20% of sudden deaths in patients with structurally normal hearts. It is 8 to 10 times more prevalent in men than in women. In a recent meta-analysis, the incidence of arrhythmic events (sustained VT or VF or appropriate implantable cardioverter defibrillator (ICD) treatment or sudden death) in patients with Brugada syndrome was 13.5% per year in patients with a history of sudden cardiac arrest, 3.2% per year in patients with syncope and 1% per year in asymptomatic patients [2]. As more data become available, the currently accepted percentages of Sudden Cardiac Death (SCD) due to BrS need to be updated to establish the real incidence of the syndrome in unexpected deaths in different populations [3].

In these last years, the research interest on BrS has significantly increased since it prevalently affects healthy young adults during their most productive years [1]. Due to its genetic component, there is a need to identify other potentially affected relatives, focusing on children [4].

There are three types of ECG changes associated with Brugada Syndrome. Type 1 shows, in more than one right precordial lead, a coved shape of the ST-segment with J wave or ST-elevation of ≥ 2 mm (mV) at its peak followed by a negative T wave with little or no isoelectric interval; in Type 2, the ST segments have a high take-off but the J amplitude of ≥ 2 mV rises to a gradually descending ST-segment remaining ≥ 1 mV above the baseline followed by a positive or biphasic T wave; these abnormalities result in a saddle-back configuration. Finally, Type 3 presents ST elevation on the right precordial leads < 1 mm (saddle-back type or coved morphology).

From a diagnostic point of view, BrS can be confirmed in non-diagnostic cases by developing the peculiar ECG patterns in V1 and V2 after administration of Na + channel blockers (i.e., ajmaline, flecainide) [5].

In addition to recognizing specific ECG patterns, genetic and environmental modulators of BrS have been identified, which play a significant role in the dynamic nature of BrS patients' ECG. In 1998 the first genetic mutation in the SCN5A (sodium voltage-gated channel alpha subunit 5) gene was associated with BrS within a family [6]. SCN5A is responsible for phase 0 of the cardiac action potential, and pathogenic variations result in the sodium channel inability to function properly. Since the identification of the first gene associated with BrS, reports of other families affected by BrS have confirmed that the disease genetic origin follows an autosomal dominant pattern of inheritance. More than 250 pathogenic variations associated with BrS have been described in 18 different genes, primarily encoded for sodium, potassium, calcium channels, or proteins associated with these channels [7]. Pathogenic variations in genes encoding desmosomal proteins have also been associated with BrS [8, 9]. Despite these ongoing developments in understanding the genetic causes of BrS, only 30–35% of clinically established cases are genetically diagnosed, and most of these (25–30%) result from pathogenic alterations in SCN5A [5]. The remaining cases may be attributable to alterations in one of the other BrS-associated genes [10]. A recent study [11] concluded that BrS ECG pattern appears not to be a pure Mendelian disorder, but rather the result of different molecular pathologies. Thus, genetic screening results do not currently influence prognosis or treatment [2], since they take into account only a part of the reported cases and variants previously classified as pathogenic may be of ambiguous significance following recent guidelines of the American College of Medical Genetics [12].

Due to disease heterogenicity in terms of genetic and phenotypic features, the use of new approaches as omics (genomics, transcriptomics, proteomics) might assume an important role in understanding the molecular mechanisms and identification of diagnostic/prognostic markers of BrS. These high-throughput technologies produce a considerable amount of data on analyzed genes, RNAs, and proteins, providing a more thorough picture of the different molecular events involved in the disease pathogenesis. In particular, omics technologies can identify new molecular mechanisms through the in-depth analysis of biological (or biochemical) pathways. This information can be extracted to differentiate BrS from similar phenocopies and develop new and reliable biomarkers for disease detection [13]. Among all techniques, the analysis of RNA profile trough transcriptomics has shown its importance in the study of several diseases [14]. This group of molecules is close to patient phenotype and can provide information on patients' condition that standard biochemical analysis or DNA variants cannot detect [15]. RNAs and their associated pathways could represent the bridge between environmental factors and genotype [16], showing the dynamic interaction during the patient's life. Among RNA molecules sub-groups, a prominent role is assumed by non-coding RNAs, particularly the micro-RNAs (miRNAs). These short coding RNA sequences can be found in the so-called microvesicles, a class of membrane vesicles ranging in size between 100 and 1000 nm in diameter, generated by outward budding or blebbing of the plasma membrane [17] during various processes and stimuli [18]. miRNAs modulate gene function by binding with specific sequences of target genes [15]. While transcription factors play a critical role in controlling ion channels/transporters at the transcriptional level, miRNAs are paramount for expression regulation at the post-transcriptional level. These small non-coding RNAs finely regulate the expression of genes. In cardiovascular disease, miRNAs analysis has assumed a leading role in several cardiovascular pathologies [19, 20].

Challenges and actual controversies do exist as far as BrS diagnosis is concerned. Although at a clinical level BrS presents specific ECG features and these criteria have a wide consensus as underlined by present guidelines [21], difficulties exist on ECG interpretation due to the extreme variability of ECG patterns that may change with time and during situations as sleep, fever and vagal stimulation [4, 5]. In contrast to diagnostic criteria for the long QT syndrome, one of the main difficulties in BrS diagnosis is the absence of exact cut-off values to base a clear-cut diagnosis.

The interpretation of these ECG patterns may vary between clinicians since different pathologies can be associated with ST-segment elevation in the right precordial leads (Table 1) [22].

**Table 1:** List of pathologies showing an ST-segment elevation in ECG patterns

Pathologies with ST-segment elevation in the right precordial leads
ST-elevation secondary to LVH
ST-elevation secondary to conduction defect (such as left bundle branch block and non-specific intracardiac conduction delay)
Normal variant of ST elevation
Early repolarization pattern (notched J-point typically in anterolateral leads)
Takotsubo (apical ballooning) syndrome
Spontaneously re-perfused STEMI
Wolf-Parkinson-White syndrome (pre-excitation)
Aneurysm/old myocardial infarction
Pericarditis/myocarditis
Hyperkalemia
Hypercalcemia

Even if specific ECG patterns are described in scientific literature, they may vary in the same individual, making the diagnosis problematic [23]. The very low prevalence of the disease associated with the high observer inter-variability in ECG interpretation and the absence of univocal "genetic makers" may lead to a reduced recognition of BrS in the population, potentially increasing risks, including sudden death.

The BrAID (Brugada syndrome and Artificial Intelligence applications to Diagnosis) project aims to integrate classic clinical guidelines for BrS diagnosis evaluation with transcriptomics approach and innovative ML algorithms for ECG pattern recognition: the combined information should lead to new diagnostic strategies in cardiovascular precision medicine of this disease.

## Methods/design

### Study design and population

The study is multicentric, non-randomized and no-profit. Patients with BrS will be enrolled at Fondazione Toscana Gabriele Monasterio, U.O.C. Cardiologia Ospedale San Donato, Azienda Usl Toscana Nord Ovest U.O.C. Cardiologia Ospedale Versilia, Azienda Ospedaliera Universitaria Careggi SOD Aritmologia, Azienda Ospedaliero Universitaria Pisana Cardiologia 2 U.O.C. Cisanello.

The protocol recognizes three phases: retrospective, prospective and validation studies. The prospective and validation studies' enrolment duration will be 24 months and will start during January 2021.

The ECG diagnosis of BrS will be strictly based on the recommendations of the 2015 European Society of Cardiology guidelines for the management of patients with ventricular arrhythmias and the prevention of SCD [2]. Inclusion criteria for BrS patients enrolment, both for the prospective and the validation study, are represented by Type 1 BrS (Coved) electrocardiographic changes, either spontaneous or induced by ajmaline/flecainide test [24, 25] or in the presence of high clinical suspicion (familiarity for BrS, patients who survived cardiac arrest with no apparent cause); age between 14 and 65 years. The control group will be represented by subjects of comparable age who undergo a cardiological examination as outpatients and present a normal baseline ECG. The presence of structural cardiac disease or concomitant diseases that may impair the completion of the protocol, lack of informed consent, pregnancy, history of coronary artery disease, severe renal or hepatic insufficiency will represent the exclusion criteria. Structural heart disease will be excluded in all patients before enrolment by non-invasive imaging (echocardiography and/or cardiac MRI).

The retrospective study aims to train the Machine Learning algorithms for BrS ECG patterns recognition; the prospective study is finalized to collect blood samples for disease-associated biomarkers by transcriptomic and microvesicles analysis, refine the ECG ML algorithm previously developed and integrate this data in the risk stratification algorithm; the validation phase will test the biomarkers discovered in the prospective study, the ECG ML algorithms and the stratification risk in a new cohort of patients.

**Fig. 1:**
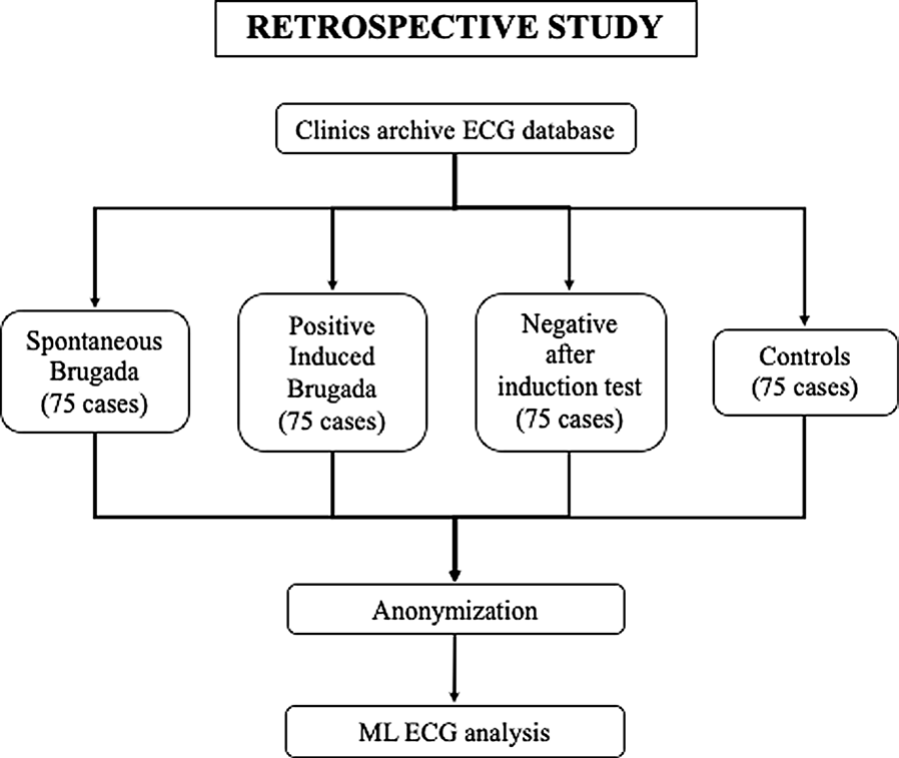
Organization of retrospective study

For the retrospective phase, the ECG traces of about 300 patients (75 with spontaneous BrS, 150 with suspected BrS and 75 controls), with standard recording parameters (speed paper 25 mm/s, amplification of 10 mm/mV, sampling rate 10 s of ECG at 500 Hz, filters: 0.5–100 Hz) will be provided in an anonymized format to the Computer Science Department of the University of Pisa for ML analysis (Fig. 1).

For the prospective phase, a new cohort of 33 patients (11 with spontaneous Type 1 BrS pattern, 11 patients in whom ECG repolarization patterns in leads V1 and V2 are suggestive of disease and a type 1 BrS pattern induced by ajmaline/flecainide infusion, 11 with suspected tracings but negative after ajmaline/flecainide test) and 11 controls will be enrolled. At baseline, each subject will undergo a cardiological examination, an ECG recording and a venous blood sampling for transcriptomics and microvesicles analysis performed.

**Fig. 2:**
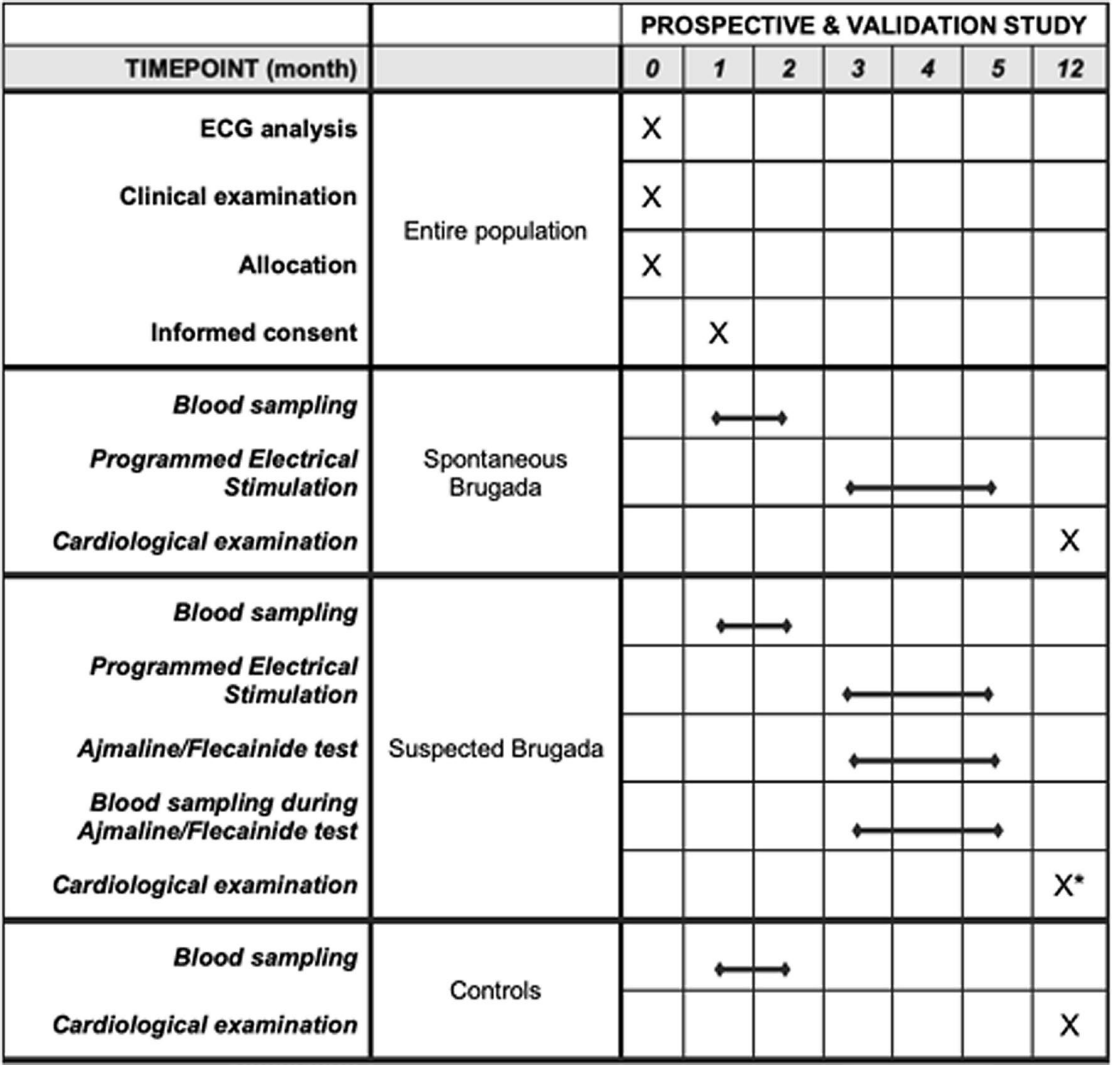
SPIRIT figure of retrospective and validation BrAID study

Subjects with either unequivocal spontaneous or pharmacologically induced ECG patterns diagnostic for Type 1 BrS will undergo electrophysiologic study (EPS) with Programmed Electrical Stimulation (PES); after 12 months a final cardiological control examination will be performed (Fig. 2).

For the validation phase a new cohort of 100 patients, of which 30 control subjects and 70 subjects (35 spontaneous BrS and 35 suspected), will be enrolled and undergo the same clinical examinations of prospective phase (Fig. 2).

The studies will be performed following the Declaration of Helsinki; they were already approved by the Comitato Etico Area Vasta Nord Ovest, Pisa, Italy. Blood tests and analyses will follow the guidelines of the Human Tissue Act (2004). The trial was registered in ClinicalTrials.gov (Identifier: NCT04641585).

### ECG

For the prospective and the validation studies, 12-lead ECG with standard recording parameters (speed paper 25 mm/s, amplification of 10 mm/mV, sampling rate 10 s at 500 Hz, filters: 0.5–100 Hz) will be acquired and the following parameters analyzed by the cardiologist: PQ interval, QRS duration, QTc interval, the presence of a Type 1 BrS pattern [25], the presence of depolarization abnormalities (major: epsilon waves; minor: terminal activation duration [TAD] ≥ 55 ms) and repolarization abnormalities (major or minor T-wave inversions) [26].

### Transcriptomics and microvesicles extraction

Whole blood will be collected in PAXgene blood RNA system tubes (DIALAB ITALIA Srl, Milan, Italy), containing reagents used to stabilize RNA immediately. This method efficiently stabilizes the intracellular RNA, preserves the sample at temperatures between − 20 and − 80 °C and allows the maintenance of the same degree of fresh blood purity. Plasma samples will be collected in polypropylene tubes containing K3 EDTA, quickly separated by centrifugation at 1500 g for 15 min at 4 °C and stored in 500 μl aliquots while serum samples will be collected in dedicated tubes (without anticoagulant) centrifuged for 15 min at 1000 g and stored at − 20 °C until use.

Total RNA will be extracted from samples collected in PAXgene tubes using a dedicated kit (PAXgene blood RNA Kit, Qiagen, Milan, Italy). Total microvesicles will be isolated from serum or plasma samples using dedicated high-efficiency kits. The miRNAs will be isolated from both blood samples and microvesicles by specific assays. All RNA samples will be stored at − 80 °C after evaluation of integrity, purity and concentration.

RNA-seq will be run using NextSeq. 500 (Illumina, San Diego, California, United States) for next-generation sequencing. The library will be prepared following the TruSeq Stranded mRNA LT kit (Illumina) protocol. Libraries will be quantified using the Qubit 2.0 fluorometer (Invitrogen, Life Technologies, Grand Island, NY) and the dimensional profile will be analyzed on the 2200 TapeStation instrument (Agilent Technologies, Santa Clara, CA).

miRNA analysis will be performed in microvesicles, and the small RNA libraries will be generated with the TruSeq Small RNA Library Preparation Kit (Illumina). Sequencing will be performed on the NextSeq 500 system (Illumina).

### Electrophysiologic study and programmed electrical stimulation (PES)

Patients with spontaneous or suspected BrS will undergo PES to assess the inducibility of ventricular tachycardia (VT) or fibrillation (VF). In according to the internationally recognized guidelines [27], PES will be performed by a cardiologist with full training in the electrophysiologic study, supported by a dedicated nurse, in the Electrophysiology Laboratory, equipped with fluoroscopy, instrumentation for recording ECG and hemodynamic signals, and equipment for resuscitation. PES will be carried out with two drive cycles (600 and 400 ms) and up to two extra stimuli [28]. The inducibility protocol would be performed from the right ventricular apex and the right ventricular outflow tract unless ventricular tachycardia was already induced at the first location.

### Ajmaline/flecainide test

These tests will be performed and interpreted by experienced cardiologists with expertise in evaluating and managing inherited cardiac conditions, with advanced cardiovascular resuscitation equipment facility. An antecubital vein will be cannulated and a continuous 12-lead ECG monitoring performed. Baseline 12 lead ECGs will be recorded after a period of rest. The ECGs and blood pressure will be assessed at 1 min intervals throughout the test. Ajmaline at a dose of 1 mg/kg will be intravenously administered as a phased infusion over 10 min [29]. For the flecainide test, the drug will be administered according to published data [30] (2 mg/kg i.v. bolus over 10 min).

The infusion will be interrupted when the ECGs becomes diagnostic with typical Type 1 BrS pattern, or the QRS duration increased by 130% or more [25]. ECGs will be monitored until the PR interval, and QRS durations are normalized. The test is considered positive if J point elevation of ≥ 2 mms with coved ST elevation in one or more right precordial leads will be recorded [31].

### Outcome measures

- Primary outcome
  The primary outcome of the BrAID study is the percentage of identification of Type 1 BrS ECG pattern with ML models compared to the diagnosis made by expert Cardiologists. Data will be obtained by the analysis of entire 12-leads ECG time series in terms of heart rate (HR), QRS interval duration, complex repolarization shape and ECG features associated with the syndrome (coved ST, QRS fragmentation, ST-segment depression, negative T wave, J wave amplitude ≥ 2 mm, broad P wave with some PQ prolongation) by the neural network in a cohort of 44 patients (prospective study) and validated in a cohort of 100 patients (validation study).
- Secondary outcomes
  Two secondary outcomes of BrAID study are: (1) the identification of biomarkers associated with Type 1 Brugada Syndrome by means of blood transcriptomic profile and microvesicles analysis of patients; (2) the development of a stratification risk system for Type 1 Brugada Syndrome by the integration of ECG Machine Learning algorithms, clinical data and biomarkers.

### Statistical analysis

For the sample calculation, reference will be made to compare sequenced RNAs, using a two-tailed Student's test for independent data. Considering the technology used to obtain RNA data [32], an expression variation of 6 (fold change) being expected, and assuming a gene coverage depth of 0.75 with a standard deviation of 1, a power of 80% and an alpha of 0.05 will be guaranteed by a sample of 11 subjects per group: 11 spontaneous BrS, 11 Suspected BrS (positive), 11 Suspected BrS (negative), 11 Control.

For bioinformatics analysis of transcriptomics, a case calling and demultiplexing will be performed raw processing data for both format conversion and demultiplexing by the Bcl2Fastq 2.20 version of the Illumina pipeline. Reads deduplication based on the unique molecular identifier (UMI) composed of 8 random bases for unambiguous identification of unique library molecules will discriminate between true PCR duplicates and independent adaptor ligation events to fragments with the same start site. DESeq2 will be used to perform comparisons between expression levels of genes and transcripts. Normalization will be performed using the median-of-ratios method [33]. An initial list of modulated genes in the current experimental set will be determined by applying a likelihood ratio test [34]. Adjusted *p*-value < 0.01 will be set an initial threshold. Further analyses on pairwise comparison will be obtained by filtration of the Fold Change (FC) calculation: the ratios between the relative expression values of two different conditions, expressed as the absolute value, will be assessed above 3.

Regarding the predictive models of Machine Learning, the sample numbers for the retrospective study (n = 300) and the final validation study (n = 100) correspond to the number of cases already classified by the clinical centers as Brugada syndrome, the former, and which can be collected during the experimental activities of the project by the same units, the latter. According to the activities foreseen in the trial, validation methodologies (cross-validation techniques) that will exploit this availability of data to develop and estimate the predictive models consistent with state of the art will be prepared [35].

## Discussion

Difficulties still hamper the recognition of Brugada syndrome: first of all, the approach to asymptomatic patients since roughly 30% of the sudden death may represent the first manifestation of the disease [36]; moreover, ECG is by itself challenging due to temporal variability of the pattern and difficulties in selecting Br patients from other pathologies.

As far as disease characterization through biochemical markers, recently interesting results on autoantibodies against cardiac and skeletal alfa-actins, keratin-24, and connexin-43 in selected BrS patients have been published [37]. However, these results need to be confirmed in larger patient groups, especially in those with clear-cut Brugada pattern after administration of Na^+^ channel blockers.

Therefore, improvement in the ECG diagnosis of Brugada syndrome through artificial intelligence techniques, the definition of new biochemical markers from omics approach, and especially the combination of these markers with a well-tailored collection of clinical data may represent the key towards an unequivocal diagnosis in subjects without structural cardiac disease at risk of sudden cardiac death.

BrAID is a study intended to investigate the role of Machine Learning models to recognize specific ECG patterns associated with Brugada Syndrome, evaluate possible novel biomolecular markers of the syndrome, and integrate these data with the clinical ones in an advanced system for patient's risk stratification.

In particular, we will evaluate ML models and blood markers' power in recognition of Type 1 BrS syndrome patients with further advancement in recognition of spontaneous respect to drug-induced patients. The study will expand the current knowledge of Brugada Syndrome and will bring additional data on this disease and its clinical implications.

### Strengths

- The present prospective multicenter study is designed to explore ML models' potential use in ECG analysis, transcriptomics, and their integration in BrS patients. It has clearly established aims, inclusion and exclusion criteria, as well as defined methods and endpoints.
- The trial is restricted to Type 1 BrS patients, excluding cardiac pathologies that could interfere with the effective recognition of diagnostic ECG patterns.
- Inclusion of positive and negative Ajmaline/Flecainide-induced BrS patterns cohorts will allow the investigation of patients with different risk profiles, supporting the ML algorithms and transcriptomics in a more effective sub-population recognition

## Limitations

- The selected sample size may be inadequate to allow a subgroup analysis.
- The BrAID study purposefully excludes patients with structural cardiac diseases and comorbidities.

### Trial status

Participant recruitment will start in January 2021. The study completion will be in September 2023.

## Data Availability

Not applicable. Study patient enrolment and data collection are currently ongoing, and no datasets were generated for analysis yet.

## Abbreviations

BrS: Brugada syndrome
ECG: Electrocardiogram
ML: Machine learning
PES: Programmed electrical stimulation
SCD: Sudden cardiac death
SCN5A: Sodium voltage-gated channel alpha subunit 5
